# Five Years of Direct Oral Anticoagulants Use in Italy: Adverse Drug Reactions from the Italian National Pharmacovigilance Network

**DOI:** 10.3390/jcm11113207

**Published:** 2022-06-04

**Authors:** Carlo Lavalle, Marco Valerio Mariani, Agostino Piro, Michele Magnocavallo, Giampaolo Vetta, Sara Trivigno, Giovanni Battista Forleo, Domenico Giovanni Della Rocca, Massimo Uguccioni, Vincenzo Russo, Francesco Summaria, Luca Di Lullo

**Affiliations:** 1 Department of Cardiovascular, Respiratory, Nephrological, Aenesthesiological and Geriatric Sciences, “Sapienza” University of Rome, Viale del Policlinico 155, 00161 Rome, Italy; marcoval.mariani@gmail.com (M.V.M.); agostino.piro@uniroma1.it (A.P.); michele.magnocavallo@uniroma1.it (M.M.); giampaolo.vetta7@gmail.com (G.V.); sara.trivigno3@gmail.com (S.T.); 2 Department of Cardiology, ASST-Fatebenefratelli Sacco, Luigi Sacco Hospital, University of Milan, 20157 Milan, Italy; forleo@me.com; 3 Texas Cardiac Arrhythmia Institute, St. David’s Medical Center, Austin, TX 78705, USA; domenicodellarocca@hotmail.it; 4 UPMC Salvator Mundi, 00152 Rome, Italy; muguccioni@scamilloforlanini.rm.it; 5 Department of Medical Transational Sciences, University of Campania “Luigi Vanvitelli”, Monaldi Hospital, 80131 Naples, Italy; v.p.russo@libero.it; 6 UOC Cardiologia, Ospedale San Eugenio, 00144 Rome, Italy; f.summaria@gmail.com; 7 Department of Nephrology and Dialysis, L. Parodi-Delfino Hospital, Piazza Aldo Moro 1, 00034 Rome, Italy; dilulloluca69@gmail.com

**Keywords:** anticoagulants, adverse drug reaction, atrial fibrillation, DOAC

## Abstract

Background: Direct oral anticoagulants (DOACs) are the preferred anticoagulant drugs for the prevention of atrial fibrillation (AF)-related thromboembolic complications and for the treatment and the prevention of recurrences of venous thromboembolism (VTE). The evaluation of self-reported adverse drug reactions (ADRs) available from databases of drug-regulatory agencies such as the Italian Medicines Agency (AIFA) pharmacovigilance database represents a novel aid to guide decision making. Objective: To assess the safety profile of DOACs by analyzing ADR rates in the real-world Italian scenario. Methods: Post-marketing surveillance data recorded by the National Pharmacovigilance Network were retrieved for the time period 2017–2021 from the AIFA online site. The following data were collected for each DOAC: total ADR number, serious ADR number, gastrointestinal (GI) ADR, intracranial hemorrhage events (ICH ADR), and more frequently reported ADR for the study year. The safety profile was expressed by the risk index (RI). Results: Rivaroxaban use was associated with consistent and stable low rates of serious ADR, GI ADR, and ICH ADR across the 5-year study period. Rivaroxaban and apixaban showed the lowest RI for serious ADR and GI ADR, while rivaroxaban use was associated with significantly lower ICH events as compared to apixaban. Dabigatran was related to the highest RIs for every ADR class, in particular GI ADRs. Conclusions: DOACs presented an acceptable safety profile in the current post-market analysis. However, rivaroxaban and apixaban were associated with more favorable safety profiles as compared to dabigatran, while rivaroxaban provoked statistically significantly fewer ICH events as compared to apixaban.

## 1. Introduction

Oral anticoagulant therapy has become pivotal in daily clinical practice due to the increasing prevalence in the general population of diseases that need anticoagulation, such as atrial fibrillation (AF) and venous thromboembolism (VTE), including both deep vein thrombosis (DVT) and pulmonary embolism (PE) [1,2]. On the one hand, atrial fibrillation is the most diagnosed clinical arrhythmia, and its prevalence has expected to increase in the next years due to the aging of the population and widespread diffusion of AF risk factors, such as chronic kidney disease (CKD), systemic arterial hypertension, obesity, heart failure (HF), diabetes mellitus (DM), and oncologic disease [3,4,5,6]. On the other hand, the former risk factors also increase the risk of VTE, which represents a leading cause of in-hospital death and requires lifelong anticoagulation if recurrent or without identified risk factors [2]. Anticoagulation therapy is pivotal in those two conditions: in AF patients, anticoagulation prevents the feared thromboembolic complications, such as ischemic stroke and systemic embolism [7], while in VTE patients’ anticoagulation clears clots preventing hemodynamic instability due to acute right HF and long-term PE sequelae such as chronic thromboembolic pulmonary hypertension (CTEPH), due to the persistence and organization of thrombi in pulmonary arterial bed [8].

Before the advent of direct oral anticoagulants (DOACs) (namely rivaroxaban, apixaban, edoxaban, and dabigatran), oral anticoagulation for prevention of thromboembolic complications related to AF or treatment of VTE was mainly based on the use of vitamin K antagonist (VKA), as warfarin. VKAs present significant practical limitations inherent to pharmacodynamic and pharmacokinetic properties that affect the efficacy and patients’ adherence to therapy, such as the need for frequent monitoring of coagulation level, common drug–drug interactions, and food–drug interactions [9]. Beyond being user friendly and not requiring coagulation monitoring, DOACs showed a favorable risk/benefit profile in terms of efficacy and safety in the AF clinical registration trials, with non-inferior or superior efficacy in preventing thromboembolic events and with non-inferior safety profile relative to VKAs [10,11,12,13]. A large meta-analysis by Ruff et al. [14], including 71,683 participants of the RE-LY, ROCKET AF, ARISTOTLE, and ENGAGE AF–TIMI 48 trials, showed that DOAC use was related to significant reductions in stroke, intracranial hemorrhage, and mortality, similar major bleeding, but increased gastrointestinal bleeding as compared to warfarin. Similarly, randomized controlled trials (RCTs) comparing DOACs with warfarin consistently demonstrated the non-inferiority of DOACs in preventing recurrent VTE at follow-up without increasing major bleeding risk [15,16,17,18]. On the basis of the results of large phase III trials and meta-analyses, current AF guidelines recommend the use of DOACs over VKAs as the preferred anticoagulant drug, excluding patients’ mechanical heart valves or moderate-to-severe mitral stenosis [19,20]. In addition, PE guidelines state that DOACs are recommended in preference to a VKA in VTE patients without contraindication [8].

However, RCTs are conducted under strict methodological conditions, including the selected population of patients that may not present the same peculiarities encountered in unselected patients with several comorbidities physicians deal with in daily clinical routine [21,22]. In this view, real-world evidence complements the results from phase III trials and is important for assessing the safety and effectiveness of approved medications and obtaining information on patterns of use in routine clinical practice. Relative to RCTs, real-life observational studies provide useful additional information, including an unselected, large, and heterogeneous population, which is more representative of patients encountered in daily clinical practice [23]. In the last years, two large post-marketing surveillance studies were published: the XANTUS program worldwide [24,25], including three prospective studies conducted in 47 different regions of the world and assessing the real-world safety profile of rivaroxaban, and the ETNA-AF program, reporting the 1-year follow-up analysis from 26,823 patients with AF across European and Asian countries taking edoxaban 60 or 30 mg doses for the prevention of stroke [26,27].

The post-marketing surveillance data on DOACs in Italy are freely available on the official portal of the Italian Drug Association (AIFA) and are based on the spontaneous report of adverse drug reactions (ADRs) by health personnel recorded in the National Pharmacovigilance Network (NPN) and available in the Adverse Reaction to Medicine (ARM) system. Two previous studies presented safety data on the use of DOACs in Italy from 2013 to 2018 [28,29]. Herein, we present a pooled analysis of ADR signaled from 2017 to 2021 with the purpose of assessing the safety profile associated with the use of DOACs in Italian daily clinical practice.

## 2. Methods

Post-marketing surveillance in Italy is regulated by a specific law released by the Ministry of Health on 30 April 2015, that stated that when a drug is suspected of having caused an ADR, the pharmacovigilance system should be activated by a spontaneous report, thus reducing the risk of under-reporting. A spontaneous report is a notification by a patient or any healthcare provider in order to alert about a possible ADR [30]. Post-marketing surveillance data recorded by the NPN were retrieved for the time period 2017–2021 from AIFA online site via the ARM system (https://servizionline.aifa.gov.it/ accessed on 31 January 2022) [31], obtaining the absolute number of confirmed ADRs reported by healthcare providers and/or consumers for each of the four molecules (rivaroxaban, apixaban, edoxaban, and dabigatran). ADRs were ulteriorly divided into serious ADR, gastrointestinal (GI) ADR, intracranial hemorrhage events (ICH ADR), and more frequently reported ADR for each molecule every year. The definitions of ADR, serious ADR, ICH ADR, GI ADR, and the methodology of searching for each of these items are available in online Supplementary Material in Tables S1–S4, respectively. Once ADR events were retrieved for each study year from 2017 to 2021, the usage rate of each DOAC expressed as market share obtained from the IQVIA (Institute for Human Data Science) was collected [32]. Eventually, we calculated the risk index (RI) for each active principle as the ratio between the serious ADR rate and the rate of usage of the molecule in the same time period [28,29]. The RI may assume all positive values from 0, and the closer its value approaches 0, the better the safety profile of the molecule, meaning the rate of ADR is low.

The aim of our study was to analyze the DOAC safety profile, reporting the rates of serious ADR, GI ADR, and ICH ADR notified during the last 5 years in Italy for each DOAC.

Data are presented as numbers and/or percentages. The chi-square test was used for the comparison of categorical variables and to seek any difference between ADR rates for each DOAC. Statistical analysis was performed using SPSS statistical software (release 26.0; SPSS Inc., Chicago, IL, USA). A *p*-value ≤ 0.05 was considered statistically significant.

## 3. Results

Database search for the time period 2017–2021 showed 6245 serious ADR associated with the use of the 4 DOACs. In particular, 30.4% (*n* = 1899) of serious ADRs was related to dabigatran, 27.1% (*n* = 1691) to apixaban, 28.2% (*n* = 1762) to rivaroxaban and 14.3% (*n* = 893) to edoxaban (Table 1). The usage rates for each DOAC during the study period are reported in Table 2. Overall, data taken from the AIFA site showed that the number of DOAC prescriptions in Italy ranged from 600,000 to 700,000/month (last data extracted on 17 January 2022). Although the monthly number of DOACs remained stable during the study period, the total number of ADR reports progressively decreased from 2017 to 2021, pointing out the effect of the under-reporting phenomenon that is likely in a system based on spontaneous reporting.

**Figure 1:**
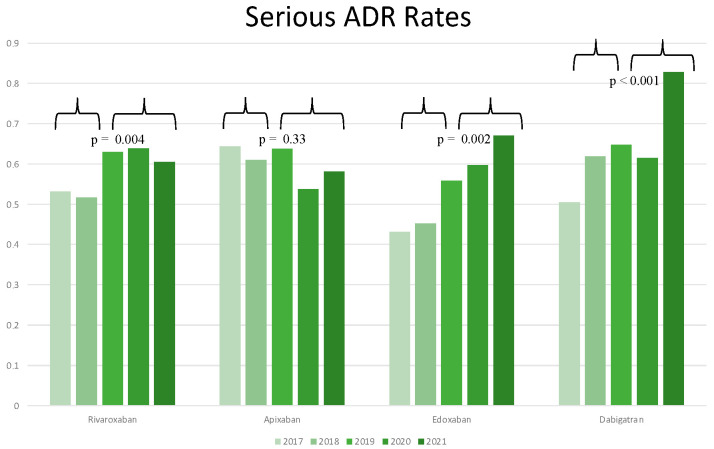
Rates of serious ADR relative to total ADR for each molecule for all study years. Serious ADR rates collected in 2017–2018 were compared to serious ADR rates collected during the period 2019–2021. A *p*-value ≤ 0.05 was considered statistically significant. ADR: adverse drug reaction.

As reported in Figure S1, there was not an overall statistically significant difference in the rate of serious ADR during the period 2017–2021 for both rivaroxaban (*p*-value = 0.07) and apixaban (*p*-value = 0.29), whereas a significant increase in serious ADR rates was recorded for edoxaban (*p*-value = 0.016) and dabigatran (*p* < 0.001). When considering the period 2019–2021 (Figure 1), the rates of serious ADR for apixaban did not significantly differ from those recorded during the period 2017–2018 (*p*-value = 0.33), while for rivaroxaban, edoxaban, and dabigatran a statistically significant increase in serious ADR rates were recorded (*p*-value = 0.004, *p*-value = 0.002 and *p*-value < 0.001, respectively). However, when comparing serious ADR rates for the period 2019–2021, no differences were found either among apixaban and rivaroxaban (*p*-value = 0.22) or edoxaban (*p*-value = 0.45 as compared to rivaroxaban and *p*-value = 0.72 as compared to apixaban), but dabigatran was associated with a significantly higher rate of serious ADR as compared to apixaban (*p*-value = 0.004) and a significantly higher rate of serious ADR as compared to edoxaban (*p*-value = 0.03).

**Figure 2:**
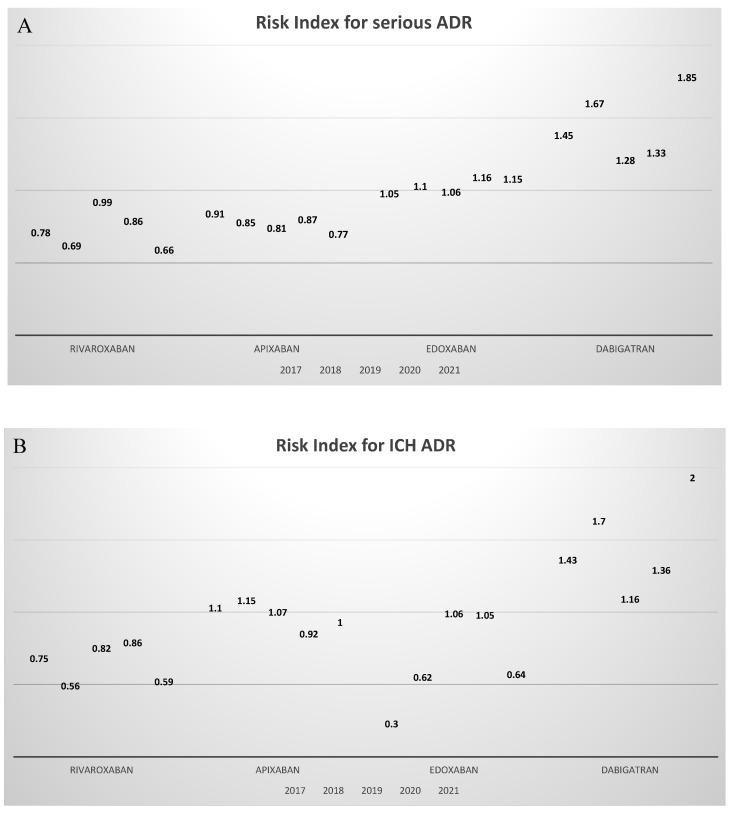

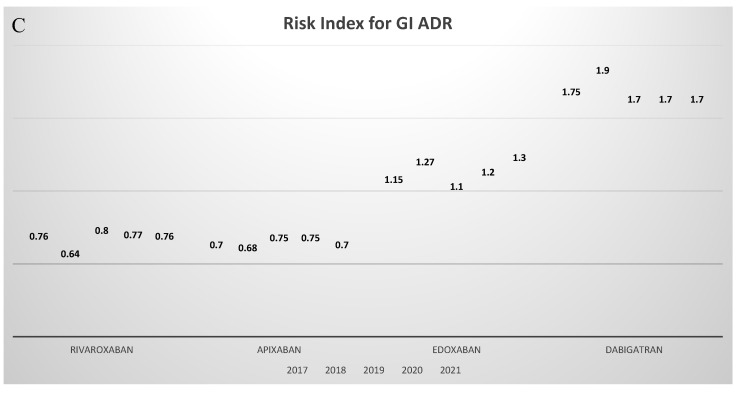
RIs for serious ADR, ICH ADR, and GI ADR for each molecule for all study years. In (**A**), RIs for serious ADR are shown. In (**B**), RIs for ICH ADR are shown. In (**C**), RIs for GI ADR are shown. ADR: adverse drug reaction; GI: gastrointestinal; ICH: intracranial hemorrhage; RI: risk index. The lower the RI, the safer the molecule. Rivaroxaban showed the lowest RI for serious ADR and ICH ADR while resulting as safe as apixaban for GI ADR. ADR: adverse drug reaction; GI: gastrointestinal; ICH: intracranial hemorrhage; RI: risk index.

Dabigatran was found to be the molecule showing the highest RI throughout the period 2017–2021, with a RI always >1, followed by edoxaban with a RI approximating 1 (Figure 2). Apixaban and rivaroxaban use was always associated with a RI < 1, indicating a safer usage profile. During the period 2019–2021, rivaroxaban was confirmed to be the molecule with a safe usage profile, with a lower RI value of 0.66 in 2021, followed by apixaban with RI of 0.77, edoxaban with RI of 1.15, and dabigatran with the highest RI of 1.85 (Figure 2A).

Nosebleed was the most common ADR reported when using rivaroxaban during the period 2017–2021, with rates ranging from 8.6% (*n* = 79) in 2019 to 13.9% (*n* = 50) in 2021 (Table 3). The most common ADRs reported when using apixaban were anemia in 2017 (6%) and 2020 (7.9%), and nosebleed in 2018 (8%), 2019 (8.6%), and 2021 (10.4%). On the same line, nosebleed and anemia were the most reported ADRs related to edoxaban use. Of note, anemia was the commonest ADR for the last 3 years in a row, and, as shown in Table 3, anemia rates increased from 9.8% in 2019 to 12.2% in 2021. GI ADRs were the commonest reported with dabigatran use. In particular, in 2018, melaena was reported in 5.9% of total ADRs, while rectal bleeding was the most common ADR in both 2019 and 2021, with rates of 9.8% and 7.8%, respectively (Table 3).

**Figure 3:**
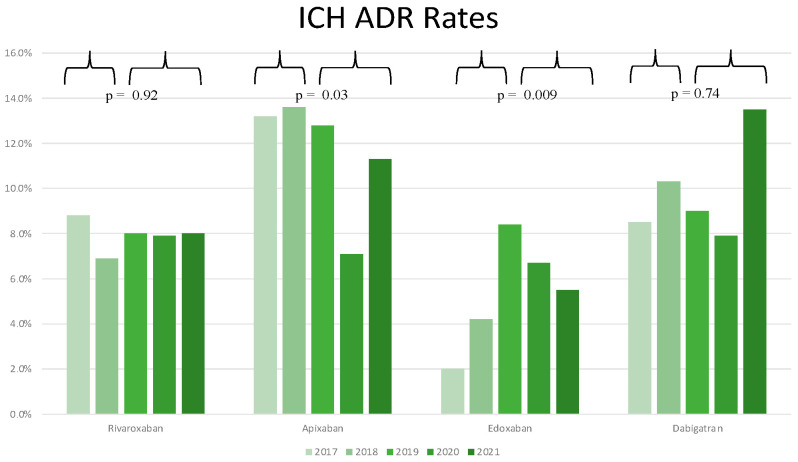
Rates of ICH ADR relative to total ADR for each molecule for all study years.

The total number of ICH events reported during the period 2017–2021 was 953. Notably, 238 events (24.9%) were related to rivaroxaban use, 323 events (33.9%) with apixaban use, 98 events (10.4%) with edoxaban use and 294 (30.8%) were associated with dabigatran usage (Table 1). As reported in Figure S2, there was not an overall statistically significant difference in the rate of serious ADR during the period 2017–2021 for both rivaroxaban (*p*-value = 0.88) and dabigatran (*p*-value = 0.12), whereas a significant difference in ICH ADR rates was recorded for edoxaban (*p*-value < 0.001) and apixaban (*p*-value = 0.009). When considering the period 2019–2021 (Figure 3), the rates of ICH ADR for rivaroxaban and dabigatran did not significantly differ from those recorded during the period 2017–2018 (*p*-value = 0.92 and *p*-value = 0.74, respectively), while for apixaban and edoxaban a statistically significant variation in ICH ADR rates was recorded (*p*-value = 0.03 and *p*-value = 0.009, respectively). Indeed, during the 2017–2021 time period, rivaroxaban was constantly associated with a low rate of ICH relative to its usage rate resulting in low RI < 1, and dabigatran was constantly associated with a high rate of ICH relative to its usage rate resulting in high RI > 1. On the other hand, edoxaban and apixaban use was associated with fluctuations in ICH RI over time, with RI above and beyond 1 in different years (Figure 2B). These results observed in a relatively long time period (5 years) underscore the reproducibility of the safety profile associated with the use of rivaroxaban as compared to the other molecules. When comparing ICH ADR rates for the period 2019–2021, no differences were found among rivaroxaban and edoxaban (*p*-value = 0.43), rivaroxaban and dabigatran (*p*-value = 0.079), or apixaban and dabigatran (*p*-value = 0.96), but rivaroxaban was associated with a significantly lower rate of ICH ADR as compared to apixaban (*p*-value = 0.022). In 2021, rivaroxaban showed the lowest RI of 0.59, followed by edoxaban with an RI of 0.64, apixaban with an RI of 1, and dabigatran with the highest RI of 2 (Figure 2).

**Figure 4:**
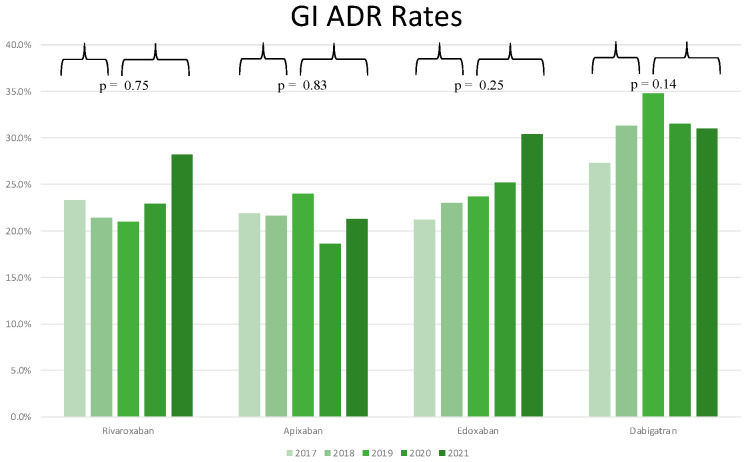
Rates of GI ADR relative to total ADR for each molecule for all study years.

During the period from 2017 to 2021, the use of dabigatran was related to 947 GI ADR (Table 1), accounting for almost 36% of total GI ADR (*n* = 2633), while rivaroxaban, edoxaban, and apixaban were associated with 25.9%, 22.9% and 15.2% of total GI ADR, respectively. There was no overall statistically significant difference in the rate of GI ADR during the period 2017–2021 (*p*-value = 0.27 for rivaroxaban, *p*-value = 0.43 for apixaban, *p*-value = 0.39 for edoxaban, and *p*-value = 0.29 for dabigatran as shown in Figure S3). No statistically significant difference was found when comparing 2019–2021 GI ADR rates with 2017–2018 rates for each molecule (*p*-value = 0.75 for rivaroxaban, *p*-value = 0.83 for apixaban, *p*-value = 0.25 for edoxaban, and *p*-value = 0.14 for dabigatran, as shown in Figure 4). When comparing GI ADR rates for the period 2019–2021, no differences were found among rivaroxaban and apixaban (*p*-value = 0.35) or rivaroxaban and edoxaban (*p*-value = 0.15). Dabigatran was associated with higher GI ADR rates as compared with the other molecules (*p*-value < 0.001 for rivaroxaban, *p*-value < 0.001 for apixaban, *p*-value < 0.001 for edoxaban). Apixaban and Rivaroxaban were associated with a RI of GI ADR < 1 during the whole period from 2017 to 2021, with a RI for the year 2021 of 0.7 and 0.76, respectively. Conversely, edoxaban and dabigatran presented a RI for GI ADR >1, with a RI for the year 2021 of 1.3 and 1.7, respectively (Figure 2).

## 4. Discussion

The present paper described the rates of serious ADR, GI ADR, and ICH ADR recorded by the pharmacovigilance system in Italy for each of the four DOACs during the period 2017–2021. The main findings of our pooled analysis are:

1. Rivaroxaban use was associated with consistent and stable low rates of serious ADR, GI ADR, and ICH ADR across the 5-year study period, while fluctuations of ADR rates were recorded for the other molecules (reduction in ICH ADRs for apixaban, increase in serious ADR and ICH ADR for edoxaban and dabigatran).
2. Rate of serious ADR for rivaroxaban, edoxaban, and dabigatran significantly increased in the period 2019–2021 relative to the period 2017–2018, while no difference was found for apixaban. When normalizing serious ADR rates for usage rates for the same time periods, rivaroxaban showed the lowest risk index for all the study periods but 2019 (RI of 0.99 for rivaroxaban and RI of 0.83 for apixaban).
3. Rate of ICH ADR for edoxaban significantly increased in the period 2019–2021 relative to the period 2017–2018, and a significant decrease was observed for apixaban, while no difference was found for rivaroxaban and dabigatran. When normalizing ICH ADR rates for usage rates for the same time periods, rivaroxaban constantly showed the lowest risk index for all the study periods, with RI as low as 0.59 in 2021.
4. Rate of GI ADR remained stable during all the study periods for all DOACs, with apixaban and rivaroxaban showing the lowest RIs.
5. Apixaban and rivaroxaban use was related to significantly lower rates of serious ADR and GI ADR as compared to dabigatran, while apixaban use resulted in a significantly higher rate of ICH ADR as compared to rivaroxaban.
6. In the last five years, nosebleed was the most common ADR reported for rivaroxaban and apixaban, and anemia was the most common for edoxaban, while rectal bleeding was the most frequent ADR reported for dabigatran.
7. Although the monthly number of DOACs remained stable during the study period, the total number of ADR reports progressively decreased from 2017 to 2021, pointing out the effect of the under-reporting phenomenon.

Although DOACs showed superiority relative to VKAs in large phase III RCTs and demonstrated to be non-inferior in terms of safety, phase IV prospective observational trials aimed at verifying the safety of drugs after being placed on the market and other real-life observational studies confirmed the safety of DOACs in a daily practice setting, also fueling the progressive expansion of DOACs indication in the subgroup of patients excluded from RCTs, such as cancer patients and end-stage renal disease patients [33,34,35,36,37,38,39,40,41,42,43,44,45,46,47]. The XANTUS program was a prospective, observational, noninterventional study evaluating the safety of rivaroxaban used for stroke prevention in 11.121 patients with AF [25]. The results of the pooled analysis showed that patients with AF throughout the world had low rates of stroke and major bleeding (1.7/100 patients-year) in the first year after initiating rivaroxaban and that fatal and critical organ bleeding events, including ICH, were rare, <0.6% of patients. Moreover, Camm et al. [48] compared the outcomes from the real-world XANTUS study with those from the Phase III ROCKET AF study with a matching-adjusted indirect comparison method, showing that the low rates of major bleeding and stroke found in the unselected population from the XANTUS were consistent with results from ROCKET AF. These safety data were also confirmed in a retrospective post-marketing analysis involving 27,647 patients, showing a remarkable low frequency of fatal bleeding (0.08/100 years/person) associated with the use of rivaroxaban [49]. The ETNA-AF program reported the 1-year follow-up analysis of 26,823 patients with AF across European and Asian countries taking edoxaban for the prevention of stroke [27]. ETNA-AF program showed a low rate of adverse events at 1-year follow-up, namely 273 (1.12%/year) major bleeding events, including 75 (0.31%/year) intracranial hemorrhages and 140 (0.57%/year) major gastrointestinal (GI) bleeds. These large programs confirm the safety of rivaroxaban and edoxaban in a real-life setting in a population with a relatively low mean HAS-BLED score of 2.

Our study confirms the safety of DOACs and expands the results of two previously published studies dealing with the use of DOACs in Italy. In 2018 Uguccioni et al. [28] firstly published Italian experience with DOACs, using data on ADR collected by AIFA in 2016–2017 and expressing the safety profile of each molecule using the RI: rivaroxaban resulted in the DOAC with a lower RI of 0.75, followed by apixaban with RI of 0.92 and lastly dabigatran with an RI of 1.5. In 2020, Lavalle et al. published updated data on ADR rates collected in 2017–2018 with DOACs use in Italy, confirming rivaroxaban as having the best safety profile with an RI of 0.69 [29]. Moreover, Lavalle et al. presented data for ICH ADRs retrieved for 2018, showing that dabigatran was the molecule with the highest RI while rivaroxaban still had the lowest RI [29]. In our update, we confirmed the results previously found, further including data on GI ADRs and on the commonest ADR for each study year. Moreover, one novelty of our study is the relatively long time of observation, from 2017 to 2021, thus allowing the evaluation of the persistence and stability of the results observed every single year. For instance, the absence of inter-year statistically significant difference in serious ADR rates for rivaroxaban and apixaban proved a reproducible safety profile in a time period of 5 years. Lastly, we performed a comparison of ADR rates among different DOACs, indicating the presence of any difference in the prevalence of ADR for each specific DOAC. Of course, no solid conclusions on what DOAC has the safest profile can be drawn by simple ADR rates comparisons. However, although the limitations of the unsolicited nature of data, the novelty of our analysis may help physicians in clinical routine decisions. Our results are in line with those found by Monaco et al. [50], who, using WHO pharmacovigilance data, showed an increased risk of GI bleeding in patients taking dabigatran and rivaroxaban, whereas apixaban was associated with an increased risk of cerebrovascular accident. Moreover, Gaio et al. [51] used correspondence analysis to uncover the relationship of suspected ADR with warfarin and DOAC use, showing that warfarin was associated with bleeding ADR, while apixaban was associated with an increased propensity of ICH ADR.

At the end of the day, DOACs proved to be safe and suitable not only as an anticoagulant strategy of choice in the healthy general population but also in patients affected by several comorbidities, similarly to those encountered in daily clinical practice. Our analysis represents a photograph of ADR rates and features in the last 5 years, confirming the results of previous studies and underscoring the favorable safety profile of DOACs. However, as shown by previous studies, each DOAC has a peculiar safety profile, and DOACs seem not to be interchangeable. Further well-designed, patient-level studies are awaited to confirm and expand our results, with the aim of tailoring anticoagulant therapy to each patient’s features and comorbidities.

## 5. Limitations

Several study limitations should be mentioned. Firstly, the lack of data on the study population makes it impossible to analyze different ADRs in relation to patient features and comorbidities that are known to be strongly related to bleeding propensity and ADR rates. Secondly, data collected from the AIFA website do not include information on possible off-label DOAC use or on molecule overdosing or underdosing, all factors that may be associated with ADRs. Thirdly, it was impossible to analyze ADR rates in relation to the different available DOAC dosages. Notably, these limitations are inherent for all DOACs and somewhat may mitigate the risk of bias when interpreting the results. The RI, though already been used in two previous publications [28,29], is not a completely validated score but is essential to normalize crude ADR rates for the usage rate of each molecule. Eventually, an important limitation of our study is the spontaneous, unsolicited nature of reporting, which inevitably leads to the under-reporting phenomenon.

## 6. Conclusions

The introduction of DOACs in clinical daily practice completely changed patients’ and physicians’ anticoagulation management, which became safer and handier as compared to the management associated with VKAs. Our observational analysis of ADR rates in Italy confirmed the favorable safety profile of DOACs. In particular, rivaroxaban and apixaban showed the safest profiles for serious ADR and GI ADR, although rivaroxaban was associated with lower rates of ICH ADR as compared to apixaban. Dabigatran was consistently associated with the highest RI for all ADR types during the whole study period.

Though several limitations, our data present a comprehensive picture of current Italian anticoagulation practice with DOACs, which will provide useful and reliable insights into real-world clinical routines. Nevertheless, present results need to be confirmed by large, prospective, well-designed RCTs.

## Figures and Tables

**Table 1:** Rates of serious ADR, GI ADR, ICH ADR for each molecule from 2017 to 2021.

Serious ADR/Total ADR	2017	2018	2019	2020	2021	Serious/Total ADR 2017–2021
Rivaroxaban	53.2% (301/566)	51.7% (304/588)	63% (582/924)	63.9% (354/554)	61% (221/362)	58.8% (1762/2994)
Apixaban	64.4% (308/478)	61% (336/551)	63.8% (444/696)	53.7% (344/641)	58.6% (259/442)	60% (1691/2808)
Edoxaban	43.2% (63/146)	45.2% (149/330)	55.9% (257/460)	69.7% (230/385)	67.1% (194/289)	55.5% (893/1610)
Dabigatran	50.5% (360/713)	61.9% (445/735)	64.8% (441/680)	61.5% (297/483)	82.8% (356/430)	62.4% (1899/3041)
Total serious ADR all DOACs/year	1032	1234	1724	1225	1030	6245
ICH ADR/Total ADR						
Rivaroxaban	8.8% (50/566)	6.9% (41/588)	8% (74/924)	7.9% (44/554)	8% (29/362)	7.9% (238/2994)
Apixaban	13.2% (63/478)	13.6% (75/551)	12.8% (89/696)	7.1% (46/641)	11.3% (50/442)	11.5% (323/2808)
Edoxaban	2% (3/146)	4.2% (14/330)	8.4% (39/460)	6.7% (26/385)	5.5% (16/289)	6.1% (98/1610)
Dabigatran	8.5% (61/713)	10.3% (76/735)	9% (61/680)	7.9% (38/483)	13.5% (58/430)	9.7% (294/3041)
Total ICH ADR all DOACs/year	177	206	263	154	153	953
GI ADR/Total ADR						
Rivaroxaban	23.3% (132/566)	21.4% (126/588)	21% (194/924)	22.9% (127/554)	28.2% (102/362)	22.7% (681/2994)
Apixaban	21.9% (105/478)	21.6% (119/551)	24% (167/696)	18.6% (119/641)	21.3% (94/442)	21.5% (604/2808)
Edoxaban	21.2% (31/146)	23% (76/330)	23.7% (109/460)	25.2% (97/385)	30.4% (88/289)	24.9% (401/1610)
Dabigatran	27.3% (195/713)	31.3% (230/735)	34.8% (237/680)	31.5% (152/483)	31% (133/430)	31.1% (947/3041)
Total GI ADR all DOACs/year	463	551	707	495	417	2633

Total number of serious ADR, ICH ADR, and GI ADR for each molecule and for each study year are also shown. ADR: adverse drug reaction; GI: gastrointestinal; ICH: intracranial hemorrhage.

**Table 2:** Usage rate (%) for each DOAC during the study period.

Study Year	2017	2018	2019	2020	2021
**Rivaroxaban**	37.5	35.6	34.4	33.4	32.4
**Apixaban**	32.6	31.7	31.6	32.2	32.7
**Edoxaban**	5.8	10.9	14	16.2	16.3
**Dabigatran**	24.1	21.8	20	18.2	18.7

**Table 3:** Most frequent ADR and ADR rate for each DOAC from 2017 to 2021.

Most Frequent ADR	Rivaroxaban	Apixaban	Edoxaban	Dabigatran
2017	Nosebleed (9.7%)	Anemia (6%)	Nosebleed (8.3%)	Abdominal pain (10.5%)
2018	Nosebleed (11%)	Nosebleed (8%)	Nosebleed (9.5%)	Maelena (5.9%)
2019	Nosebleed (8.6%)	Nosebleed (8.6%)	Anemia (9.8%)	Rectal bleeding (9.8%)
2020	Nosebleed (12.8%)	Anemia (7.9%)	Anemia (9.7%)	Anemia (7.4%)
2021	Nosebleed (13.9%)	Nosebleed (10.4%)	Anemia (12.2%)	Rectal Bleeding (7.8%)

ADR: adverse drug reaction.

## Data Availability

Study data are freely available at https://servizionline.aifa.gov.it/ (accessed on 31 January 2022).

## Supplementary Materials

The following supporting information can be downloaded at: www.mdpi.com/article/10.3390/jcm11113207/s1, Figure S1. Rates of serious ADRs relative to total ADR for each molecule for all study years. Figure S2. Rates of ICH ADRs relative to total ADRs for each molecule for all study years. Figure S3. Rates of GI ADRs relative to total ADR for each molecule for all study years. Table S1. Adverse reaction to Medicine system of the National Pharmacovigilance Network: definitions of adverse reaction and serious adverse drug reaction. Table S2. Definitions used to search for intracranial hemorrhage (ICH) adverse drug reactions. Table S3. Definitions used to search for gastrointestinal (GI) adverse drug reactions. Table S4. How to search for adverse drug reactions (ADRs).
