# Supplementary material for: Five Years of Direct Oral Anticoagulants Use in Italy: Adverse Drug Reactions from the Italian National Pharmacovigilance Network
Source: J Clin Med. 2022 Jun 4;11(11):3207. doi: 10.3390/jcm11113207 (PMC9181424; doi:10.3390/jcm11113207)
Supplement: Supplementary file 1 [file jcm-11-03207-s001.zip › jcm-1738517-supplementary.pdf]

Table S1. Adverse reaction to Medicine system of the National Pharmacovigilance Network: definitions of adverse reaction and serious adverse drug reaction.

Adverse drug reaction definition<br><br>By adverse reaction it is intended a harmful and unintended effect consequent not only to the authorized use of a drug under normal conditions of use, but also to therapeutic errors and uses not complying with authorized indications, including misuse and abuse of the drug.
Serious adverse reactions include:<br><br>1. a fatal reaction;<br>2. a reaction that caused or prolonged hospitalization;<br>3. a reaction that has endangered the patient's life;<br>4. a reaction that has caused serious and permanent disability;<br>5. a reaction that caused congenital anomalies and/or birth defects;<br>6. a reaction that is included in the European list of Important Medical Events (IME)
The health worker can however define a serious reaction even if the conditions described above are not present, based on the relevance of the event.<br>All other situations (not included among those listed) are to be considered as not serious.
Source: Italian Drug Agency; http://www.agenziafarmaco.gov.it/content/online-i-dati-sulle-segnalazioni-di-sospette-reazioni-avverse-registrate-nella-rete-nazional

Table S2. Definitions used to search for intracranial hemorrhage (ICH) adverse drug reactions.

Cerebral hemorrhage
Subarachnoid hemorrhage
Intracranial hemorrhage
Cerebellar hemorrhage
Intraventricular hemorrhage
Thalamic hemorrhage
Brain stem hemorrhage
Subdural hemorrhage
Subdural hematoma
Cerebral Hematoma
Subarachnoid hematoma
Cerebellar hematoma
Intracranial hematoma
Extradural hematoma
Cerebral edema
Hemorrhagic stroke
Hemorrhagic cerebral infarction
Basal ganglia hemorrhage

Table S3. Definitions used to search for gastrointestinal (GI) adverse drug reactions.

Angiodysplasia
Hematemesis
Anal bleeding
Oral bleeding
Large bowel bleeding
Small bowel bleeding
Inferior gastrointestinal bleeding
Bowel bleeding
Rectal bleeding
Duodenal ulcer bleeding
Gastric ulcer bleeding
Upper gastrointestinal tract bleeding
Gastric hemorrhage
Gastroduodenal hemorrhage
Gastrointestinal hemorrhage
Rectal hemorrhage
Anal hemorrhage
Hemorrhoidal bleeding
Hemorrhagic gastritis
Erosive hemorrhagic gastritis
Melena
Bowel adenoma bleeding
Hemorrhagic intestinal diverticulum
Pharynx bleeding
Hemorrhagic diarrhea
Tongue bleeding

Table S4. How to search for adverse drug reactions (ADR)

The ADR search can be performed:<br><br>1. By trade name of the drug indicated as suspect in the report;<br>2. By name of the active ingredient or associations of active ingredients indicated as suspicious in the report (in this way the system will add all the reports related to drugs containing that or those active ingredients).
The search results are displayed in 5 screen outputs:<br><br>1. the first one provides the total number of reports recorded in the National Surveillance Network divided by year; in the following screen outputs, the year has to be selected select from the drop-down menu at the top right;<br>2. the second provides the number and rate of reports by severity level;<br>3. the third provides the number and rate of reports divided by sex and age group of the subjects who have shown the adverse reaction(s);<br>4. the fourth provides the number and rate of ADRs aggregated by apparatus or organ concerned (SOC-System Organ Classification);<br>5. the fifth provides the number and the ADR rate aggregated in more detail (PT-Preferred Term).
The total of the ADRs can be equal to or greater than the total of the reports, as within each report it is possible to describe one or more ADRs
Source: Italian Drug Agency; http://www.agenziafaraco.gov.it/content/online-i-dati-sulle-segnalazioni-di-sospette-reazioni-avverse-registrate-nella-rete-nazional

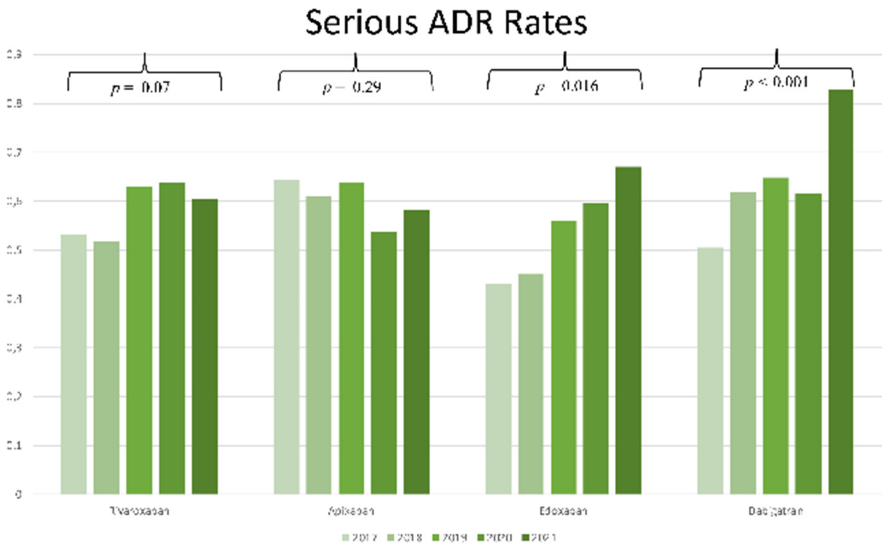

p-value for the overall comparison of serious ADR rates during the whole study period (2017-2021) are shown. In A *p*-value ≤ 0.05 was considered statistically significant. ADR: adverse drug reaction.

Figure S1. Rates of serious ADR relative to total ADR for each molecule for all study years.

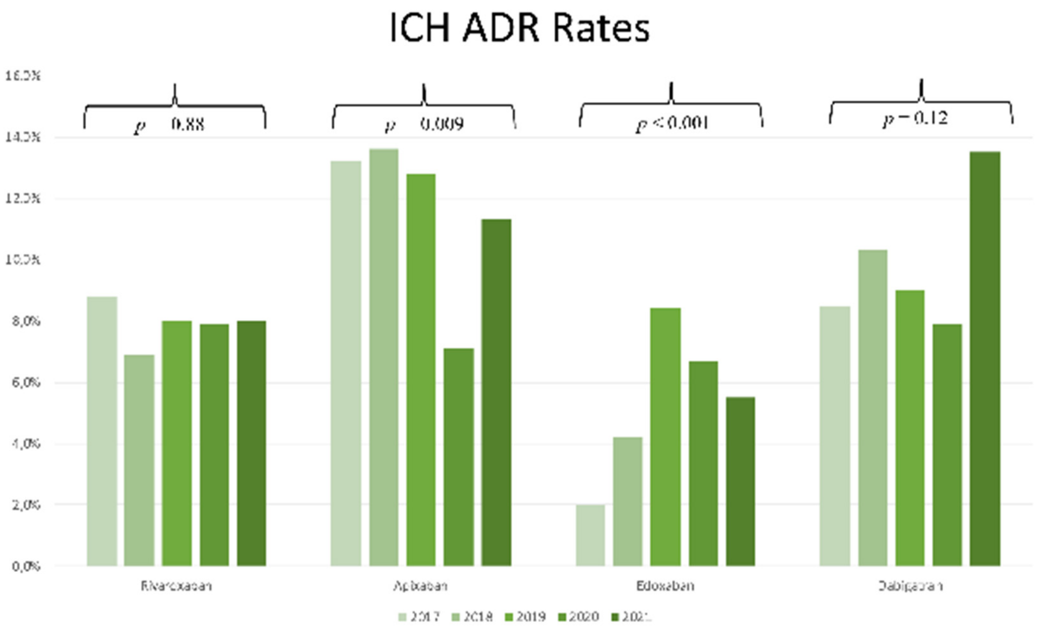

p-value for the overall comparison of ICH ADR rates during the whole study period (2017-2021) are shown. In A *p*-value ≤ 0.05 was considered statistically significant. ADR: adverse drug reaction; ICH: intracranial hemorrage.

Figure S2. Rates of ICH ADR relative to total ADR for each molecule for all study years.

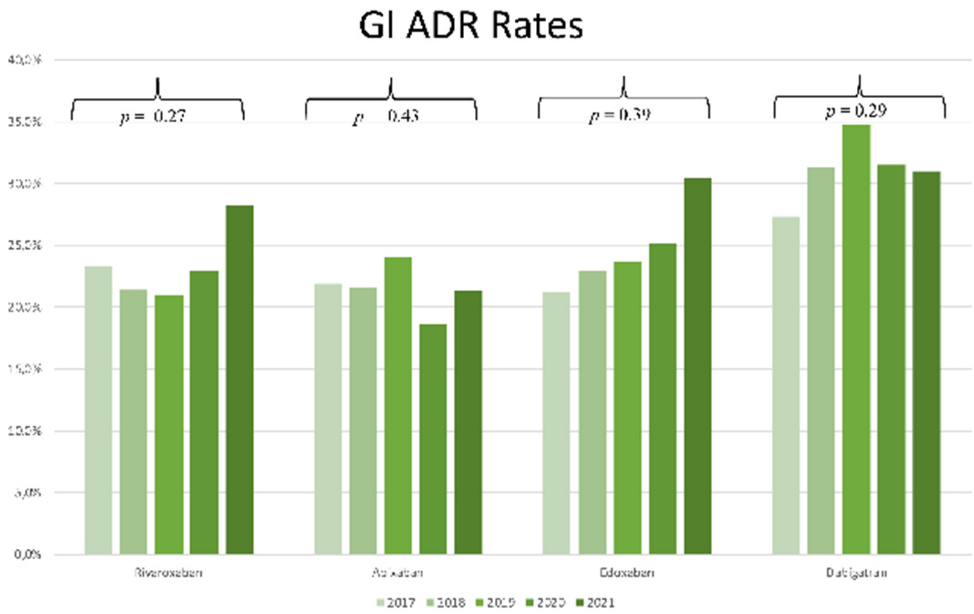

p-value for the overall comparison of GI ADR rates during the whole study period (2017-2021) are shown. A *p*-value 0.05 was considered statistically significant. ADR: adverse drug reaction; GI: gastrointestinal

Figure S3. Rates of GI ADR relative to total ADR for each molecule for all study years.
